# Global health, climate change and migration: The need for recognition of “climate refugees”

**DOI:** 10.7189/jogh.13.03011

**Published:** 2023-03-24

**Authors:** Saverio Bellizzi, Christian Popescu, Catello M Panu Napodano, Maura Fiamma, Luca Cegolon

**Affiliations:** 1 University of Sassari, Sassari, Italy; 2 University Medical Center Goettingen, Goettingen, Germany; 3 Clinical-Chemical Analysis and Microbiology Laboratory, “San Francesco” Hospital, Nuoro, Italy; 4 University of Trieste, Department of Medical, Surgical & Health Sciences, Triste, Italy; 5 Occupational Medicine Unit, University Health Agency Giuliano Isontina (ASUGI), Trieste, Italy

Climate change is considered to be the greatest threat to public health in the coming decades, as ensuing environmental variations lead to population shifts [1].

In June 2022, the number of displaced people worldwide reached an all-time high at over 100 million [2]. Although they are temporary, weather-related disasters are increasingly becoming a major cause of displacements globally; according to the United Nations High Commissioner for Refugees (UNHCR), they have caused approximately 21 million displacements annually since 2008 [3,4]. The number of weather-related disasters almost tripled in the past 40 years, with their frequency and intensity exacerbated by climate change [2]. According to the “Groundswell – Preparing for Internal Climate Migration” World Bank report, without urgent national and global climate action, South Asia, sub-Saharan Africa, and Latin America could witness more than 140 million people move within their countries’ borders by 2050 [5]. Such figures are expected to surge in coming decades and the Institute for Economics & Peace (IEP) predicts that around 1.2 billion people could be displaced by 2050 due to natural disasters and climate change [6].

Climate refugees are defined as “forgotten victims of climate change” for several reasons, first due to the lack of any data [7]. The information we do have indicates, for instance, that the number of people living in coastal areas at high risk of rising sea levels has increased from 160 million to 260 million over the past 30 years [7]. Additionally, nine out of ten of those people are from poor developing countries and small island states [6,7]. Bangladesh is a striking example of this, where 20 million people are predicted to lose their homes by 2050, as 17% of the country will be submerged due to rising sea levels [7].

The climate crisis is affecting individuals and global public health systems in almost every region of the world. This is particularly evident in the rising cases of malnutrition due to droughts and the increasing incidence of water-borne diseases such as cholera due to floods [8,9], which negatively affects already weakened and overstretched health systems, particularly in low- and middle-income countries [8,10,11].

Refugees and migrants have specific physical and mental health needs linked to their exposure to climate and environmental conditions, and their vulnerability must be considered in a whole-of-route approach [12]. They have a relevant number of health risks before, during, and after their journeys, with access to primary care and continuity of health care often disrupted due to migration, weak health system capacity, barriers (eg, gender, cultural, financial, social, and linguistic), and several other stressors, including abuse and exploitation [8,13-15].

Since 1985, experts from the United Nations Environment Programme (UNEP) have been using the term “climate refugees” for people who have been “forced to leave their traditional habitat, temporarily or permanently, because of marked environmental disruption” [16]. However, unlike war or persecution, people typically cannot claim asylum based on climate change reasons alone [17].

The Global Compact on Safe, Orderly and Regular Migration [18], adopted by most UN Member States in 2018, states that governments in receiving countries should work to protect climate refugees by devising planned relocation and visa options if adaptation and return to their countries of origin is not possible. During the same year, the UN Human Rights Council found that many people forced from their homes due to the effects of climate change do not fit the definition of refugees, ie, a person who has crossed an international border “owing to well-founded fear of being persecuted for reasons of race, religion, nationality, membership of a particular social group or political opinion”, labeling them “the world’s forgotten victims” [19,20]. This was problematic, as a declined refugee status means that, for example, such individuals have only limited access to legal protections of their human rights and face risks like deportation.

**Figure:**
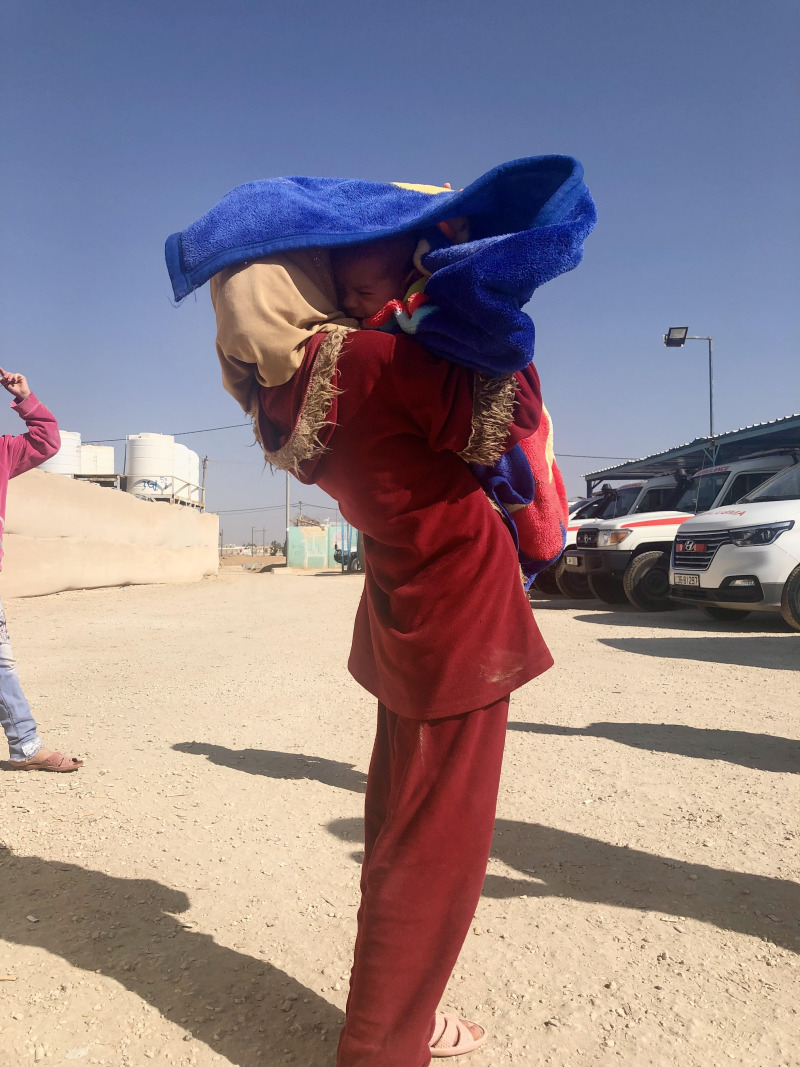
Photo: Syrian Refugees in Jordan. From Saverio Bellizzi’s own collection.

In 2020, UNHCR updated its guidance, making a broader case for the protection of those facing environmental risks [21]. In 2022, Argentina created a special visa for persons displaced by natural disasters; similarly, Finland is exploring the option to accept refugees on climate grounds. Also, Australia is introducing a scheme that will make it easier for Pacific Islanders (one of the populations most vulnerable to climate change) to move there for seasonal work. Similar measures are being discussed elsewhere [17], and their spread and the intense discussions around them indicate an increasing awareness of climate refugees and visa opportunities.

While it is too early to assess the outcomes of these initiatives, their recent spread and the discussions evolving around them highlight the growing awareness and need to formally recognize the status of climate refugee by developing structured pathways for regular migration, as well as mitigation and adaptation strategies. All relevant stakeholders, especially countries of the Global North, should uphold human rights by supporting adaptation and mitigation strategies and by harnessing the potential of climate mobility. The rational for this is simple: climate change, largely driven by the Global North, is forcing people to flee, thus limiting their access to human rights like the right to health. It is therefore the responsibility of the Global North to make sure access to those rights is upheld or restored.

Particular attention should be focused on investing in early warning systems and preparedness, facilitating a just transition to environmentally sustainable, green economies and societies (including by building climate-resilient health systems), preventing and addressing situations of vulnerability (such as promoting inclusion in immunization initiatives [22]), and fostering evidence-based decisions and disaggregated data which inform cooperation and scaled-up climate action.
